# Association between the body mass index, waist circumference, and body fat percentage with erosive esophagitis in adults with obesity after sleeve gastrectomy

**DOI:** 10.12688/f1000research.106723.1

**Published:** 2022-02-22

**Authors:** Alba S. Zevallos-Ventura, Gabriel de la Cruz-Ku, Fernando M. Runzer-Colmenares, Jesús Pinto-Elera, Carlos J. Toro-Huamanchumo

**Affiliations:** 1 Universidad cientifica del sur, Lima, Lima, Peru; 2 CHANGE Research Working Group, Universidad Científica del Sur, Lima, Lima, Peru; 3 General Surgery, Mayo Clinic in Rochester, Rochester, Minnesota, USA; 4 General Surgery, University of Massachusetts, Worcester, Massachusetts, USA; 5 Unidad de Investigación Multidisciplinaria, Clínica Avendaño, Lima, Lima, Peru; 6 Unidad para la Generación y Síntesis de Evidencias en Salud, Universidad San Ignacio de Loyola, Lima, Lima, Peru

**Keywords:** Obesity, Esophagitis Peptic, Abdominal fat, Body mass index, Waist circumference

## Abstract

**Background:** High anthropometric indexes before sleeve gastrectomy (SG) are associated with an increased risk of erosive esophagitis (EE) in bariatric surgery candidates. Reasons that explain how these indexes influence the development of esophageal pathology after surgery remains unclear.

**Objectives:** To assess the association between the body mass index (BMI), waist circumference (WC), and body fat percentage (BFP) with the development of EE in adults with obesity three months after SG.

**Setting:** Clínica Avendaño, Lima, Peru.

**Methods:** Retrospective cohort using a database including adults with obesity who underwent SG during 2017-2020. All the patients included had an endoscopy before and after the surgery. Sociodemographic, clinical and laboratory characteristics were compared according to BMI, WC and BFP, as well as by the development of *de novo* esophagitis. The association was evaluated by crude and adjusted generalized linear models with the log-Poisson family.

**Results:** From a total of 106 patients, 23 (21.7%) developed EE. We did not find significant differences in sociodemographic, clinical and laboratory characteristics between patients with de novo EE compared to those who did not develop EE. After adjustment, BMI (aRR = 0.59, 95% CI = 0.18-1.40), BFP (aRR = 0.41, 95% CI = 0.15-1.19) and WC (aRR = 0.91, 95% CI = 0.69-1.16) were not associated with the development of EE three months post SG.

**Conclusions:** We found no association between preoperative anthropometric indexes and the development of *de novo* EE; therefore, morbid obesity should not be a criterion to exclude the patients to undergo SG as primary surgery because of the risk of developing EE.

## Introduction

Obesity is currently considered as a chronic and multifactorial metabolic-related disease, of which prevalence has been increasing along the decades, ^1^ and has an important impact on morbidity and mortality worldwide. ^2^^,^^3^ Surgical treatment is available and is managed with different techniques by bariatric and minimally invasive procedures. To date, sleeve gastrectomy (SG) is the most commonly used technique ( American Society for Metabolic and Bariatric Surgery). ^4^^,^^5^

There is a direct relationship between obesity and the development of gastroesophageal reflux disease (GERD). In fact, the elevated intra-abdominal pressure and the increased transient lower esophageal sphincter (LES) relaxation ^6^ in patients with obesity has been described as a pathophysiological mechanism of GERD, and, consequently, esophageal mucosal damage ^7^ which leads to erosive esophagitis (EE). ^8^ Moreover, there are several studies that associate high values of body mass index (BMI), ^9^ waist circumference (WC), abdominal subcutaneous fat, and visceral fat ^10^^–^^12^ with the esophageal pathology. However, in a Swedish community-based study, Lagergren J *et al*. concluded that this parameter is not a sufficient indicator and would even qualify as inaccurate as a predisposing factor for EE. ^13^ Furthermore, a study among an Iranian population showed that the symptoms of GERD occur independently of the BMI. ^14^

There is scarce literature that explores the association between anthropometric indexes and the development of *de novo* EE in gastrectomized patients in a short-term period; Jan S. Burgerhart showed that esophageal acid exposure increased significantly when comparing 24-h pH measurements before and three months after sleeve gastrectomy. ^15^ Furthermore, because the rate of de novo EE is higher after SG compared to gastric bypass ^16^^,^^17^ evaluating the effect of BMI, WC and body fat percentage (BFP) on the development of *de novo* EE might be invaluable to predict whether patients with high anthropometric indexes can undergo this type of procedure. Hence, we sought to determine the association between BMI, WC and BFP with *de novo* EE three months after undergoing sleeve gastrectomy.

## Methods

### Population and study design

We conducted a retrospective cohort study, analyzing a secondary database to which we had access between January and March 2021. The database was recorded in an Excel 2016 spreadsheet of the Clínica Avendaño that was collected between 2017 and 2020 from patient medical records. The study population consisted of 176 adults with obesity that underwent sleeve gastrectomy as primary surgery during 2017-2020 at the Clínica Avendaño, a specialized bariatric center located in Lima, Perú

**Figure 1.**
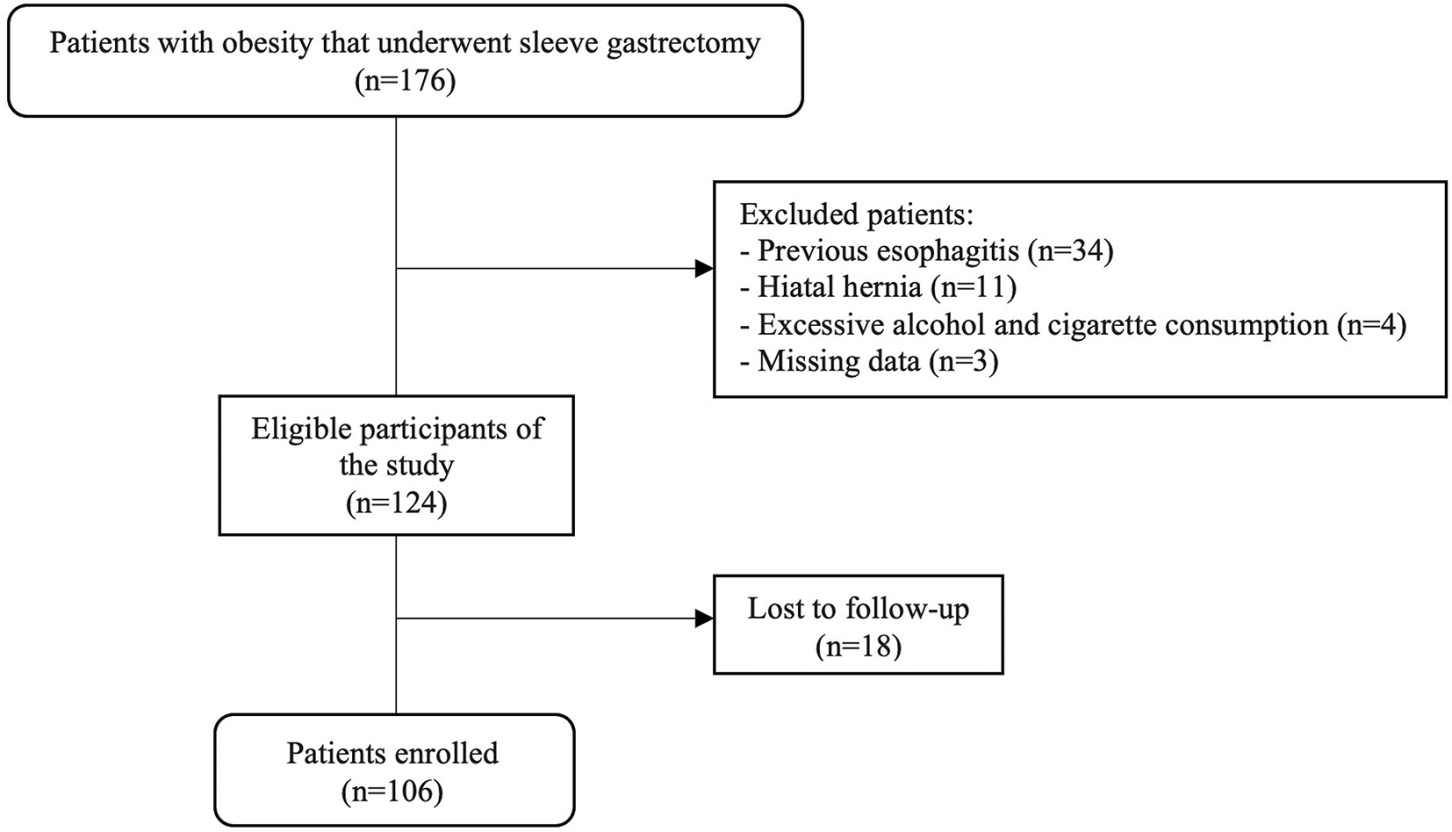
Flowchart of the study population.

The inclusion criteria were: age ≥18 years old, BMI ≥30kg/m ^2^, sleeve gastrectomy as primary surgery, and endoscopy performed preoperatively and three months post SG. The exclusion criteria were: esophagitis at preoperative endoscopy, diagnosis of hiatal hernia, excessive alcohol consumption (chronic and periodic alcohol consumption of more than three times per week) and heavy smoking (15 cigarettes or more per day). In addition, we excluded patients with missing data, as well as those who were lost to follow-up, which took place in the third postoperative month at Clínica Avendaño where a control endoscopy was performed. ( Figure 1).

### Variables and measurements

We considered demographic variables (age, sex), comorbidities (type 2 diabetes mellitus (T2DM), hypertension), clinical and laboratory variables (systolic blood pressure (SBP), diastolic blood pressure (DBP), cholesterol, triglycerides, low-density lipoprotein (LDL), high-density lipoprotein (HDL), very low-density lipoprotein (VLDL), glucose, insulin, homeostatic model assessment of insulin resistance (HOMA-IR), anthropometric variables (BMI, WC, percentage of BFP) and endoscopic variables (presence of *Helicobacter pylori* and *de novo* esophagitis at three months)). Blood analyses after fasting for 8 to 12 hours were performed for preoperative control in all patients at approximately 2 to 4 weeks before surgery (COBAS 60000 module C501).

Age was categorized into two groups (18-29 and 30-59 years) while the sex variable was defined as "male" or "female". The BMI was calculated as weight (in kg) /height (in meters) ^2^ and was categorized into non-morbid (<40.00 kg/m ^2^) and morbid obesity (≥40.00 kg/m ^2^). WC was measured between the lowest ribs and the iliac crest, and the measurements were recorded in centimeters. ^18^ BFP was recorded as a percentage using the ¨TANITA¨ bioelectrical impedance scale (Body Composition Analyzer TBF-310GS) with cut-off points of 25% for men and 35% for women categorized as normal and elevated. ^19^ The HOMA-IR was calculated as follows: [fasting insulin (μU/ml) × fasting glucose (mg/dl)] /405. A cut-off ≥ 2.5 was considered as insulin resistance. ^20^

### Procedures

Esophagitis was endoscopically evaluated prior to surgery and at three months after the procedure for each patient. The degree of esophagitis was classified according to the Los Angeles classification system and subsequently categorized as presence or absence of esophagitis. ^21^ The endoscopies were performed after preparation of the patient with a standardized technique using a flexible endoscope (OLYMPUS EXERA 180). The presence of *H. pylori* was considered if at least 1+ was observed in the preoperative gastric biopsy report. All surgical procedures were performed by the same physicians using a standardized technique that consists of dissection of the greater omentum until the complete visualization of the left pillar of the diaphragm, liberation of the posterior gastric wall, and dissection of the diaphragmatic crura. In case of finding hiatal hernia the correction of the hernia is performed intraoperatively, a 34F calibration bougie is used, the section is 4 cm from the pylorus until 1 cm from his angle. In all patients, reinforcement is performed with absorbable monofilament suture or with staples with polyglycolic acid reinforcement material.

### Statistical analysis

We calculated the statistical power with Epidat v4.2 according to the studies of Tai *et al* ^22^ and Matar *et al.* ^23^ For all scenarios the statistical power exceeded 90%.

Stata version 16.0 (StataCorp, TX, US) was used for data processing. Numerical variables were presented as mean and standard deviation or median and interquartile range. Categorical variables were presented as frequencies and percentages. The chi-square test was used to compare frequencies between groups; if more than 20% of the expected values were ≤5, the Fisher exact test was used instead.

We performed a first bivariate analysis using the Student’s t-test or Mann Whitney U test to evaluate the presence of significant differences between the anthropometric indexes and categorical variables depending on the normal and abnormal distribution of the variable, respectively. Moreover, Spearman correlation was used for assessing the relation between the anthropometric indexes and numerical variables. In addition, a second bivariate analysis was performed between numerical and categorical variables according to the presence of EE.

Finally, in order to assess the association between each index and *de novo* EE, individual generalized linear models (GLM) with the Poisson family, logarithmic link function, and robust variances were used. The WC variable was modeled by adding a quadratic term. Nonparametric bias-corrected and accelerated bootstrap estimation of confidence intervals with 1000 replications were performed for all the models. Crude relative risks (cRR) and adjusted relative risks (aRR) were calculated for the bivariate and multivariable analyses. In addition, these models were adjusted by age and sex. All models were presented with their respective 95% confidence intervals (95% CI), and a p-value <0.05 was considered significant.

### Ethical considerations

The present study was approved by the Ethics Committee of the Universidad Científica del Sur on December 1 ^st^, 2020 (N°405-2020-PRE15). We downloaded deidentified information from the database and used codes for each patient, maintaining the confidentiality of the patients.

## Results

A total of 106 patients were included in our study ( Figure 1), of which 74 (69.8%) were women, 76 (71.7%) were between 30 and 59 years of age and 32 (30.2%) had morbid obesity. A higher mean of BMI was observed in males (40.28 kg/m ^2^ vs. 36.85 kg/m ^2^; p < 0.001) and patients with insulin resistance (38.7 kg/m ^2^ vs. 33.87 kg/m ^2^; p < 0.001). Regarding BMI, a negative monotonic correlation was found with HDL (r= -0.35; p < 0.001); on the other hand, a positive monotonic correlation was found with insulin (r= +0.59; p<0.001), and HOMA-IR (r= +0.61; p<0.001). Regarding the BFP, males presented a higher median value compared to females (50% vs. 43%; p<0.001), as well as a positive correlation with SBP (r= +0.36; p<0.001), insulin (r= +0.47; p<0.001) and HOMA-IR (r= +0.48; p<0.001). Regarding WC, we found higher median values in male patients compared to females (126cm vs. 104cm; p<0.001), and in patients with T2DM (134.33cm vs. 111.51cm; p<0.001), presence of *H. pylori* (119.61cm vs. 109.29cm; p<0.001) and insulin resistance (115.31cm vs. 100.52cm; p<0.001). In addition, there was a negative monotonic correlation with HDL (r= -0.44; p<0.001), and a positive monotonic correlation with glucose levels (r= +0.31; p < 0.001), insulin (r= +0.58; p < 0.001) and HOMA-IR (r= +0.61; p<0.001) ( Table 1 and 2).

**Table 1.** Body mass index, body fat percentage and waist circumference according to sociodemographic, comorbidities, presence of *Helicobacter pylori*, insulin resistance and *de novo* esophagitis (n=106).

Variables		BMI	p	Body fat mass	P	Waist circumference	p
Age			0.621 ††		0.641 ††		0.104 †
	18-29	36 [34-41]		43 [36-5]		108.43 ± 14.33	
	30-59	38 [33-41]		46 [37-52]		114.52 ± 18.19	
**Sex**			**<0.001** **†**		**<0.001** **††**		**<0.001** **††**
	Male	40.28 ± 5.51		50 [44-58]		126 [115-134]	
	Female	36.85 ± 5.40		43 [36-50]		104 [96-115]	
**Arterial hypertension**			0.041 ††		0.019 ††		0.009 ††
	No	37 [33-41]		44 [36-51]		108 [98-120]	
	Yes	39 [37-41]		48 [43-58]		117 [111-132]	
**Diabetes mellitus**			0.021 ††		0.015 ††		**<0.001** **†**
	No	37 [33-41]		45 [37-52]		111.51 ± 16.76	
	Yes	41 [40-41]		59 [46-82]		134.33 ± 12.61	
***Helicobacter pylori***			0.019 ††		0.201 ††		**<0.001** **†**
	No	36 [33-40]		44 [36-53]		109.29 ± 17.16	
	Yes	39 [35-41]		47 [41-56]		119.61 ± 15.79	
**Insulin resistance**			**<0.001** **†**		0.024 ††		**<0.001** **†**
	No	33.87 ± 3.06		38 [36-47]		100.52 ± 9,17	
	Yes	38.70 ± 5.70		46 [38-56]		115.31 ± 17.58	
**de *novo* esophagitis**			0.264 ††		0.186 ††		0.119 ††
	No	38 [34-41]		46 [37-55]		113 [100-123]	
	Yes	36 [33-39]		44 [35-48]		107 [95-116]	

BMI body mass index

^†^ Student's t-test

^††^ Mann Whitney U

**Table 2.** Body mass index, body fat percentage and waist circumference according to clinic and laboratory characteristics (n=106).

Variables	BMI	p	Body fat mass	P	Waist circumference	p
**Clinic and laboratory characteristics**						
SBP	0.25	0.010 ‡	0.36	**<0.001** **‡**	0.27	0.006 ‡
DBP	0.2	0.039 ‡	0.26	0.006 ‡	0.2	0.038 ‡
Glucose	0.23	0.019 ‡	0.16	0.100 ‡	0.31	**<0.001** **‡**
Cholesterol	-0.15	0.114 ‡	-0.1	0.300 ‡	-0.12	0.205 ‡
HDL	-0.35	**<0.001** **‡**	-0.27	0.005 ‡	-0.44	**<0.001** **‡**
LDL	-0.06	0.532 ‡	-0.06	0.557 ‡	-0.01	0.956 ‡
VLDL	0.09	0.351 ‡	0.17	0.089 ‡	0.16	0.106 ‡
Triglycerides	0.09	0.321 ‡	0.18	0.069 ‡	0.17	0.077 ‡
Insulin	0.59	**<0.001** **‡**	0.47	**<0.001** **‡**	0.58	**<0.001** **‡**
HOMA-IR	0.61	**<0.001** **‡**	0.48	**<0.001** **‡**	0.61	**<0.001** **‡**

BMI body mass index, SBP systolic blood pressure, DBP diastolic blood pressure HDL high-density lipoprotein, LDL low-density lipoprotein, VLDL, very low-density lipoprotein, HOMA-IR homeostatic model assessment-insulin resistance

^‡^ Spearman's correlation coefficient

We found that 23 patients (21.7%) developed esophagitis (grade A: 14, grade B: 9, grade C:0, grade D:0). There were no significant differences for the sociodemographic, clinic and laboratory characteristics according to the development of EE after SG. In spite of these results, the group which did not develop EE had a higher median BMI (38 kg/m ^2^ vs. 36 kg/m ^2^, p=0.26), WC (113cm vs. 107cm, p=0.12), and BFP (46% vs. 44%, p=0.18) compared to the group who developed EE ( Table 3).

**Table 3.** *De novo* esophagitis according to sociodemographic,clinic and laboratory characteristics, comorbidities, presence of *Helicobacter pylori* and anthropometric characteristics (n=106).

Variables	*de novo* esophagitis		
	Yes (n=23)	No (n=83)	p
**Age n(%)**			0.500 ^ǂ^
18-29	6 (20.0)	24 (80.0)	
30-59	17 (22.4)	59 (77.6)	
**Sex**			0.500 ^ǂ^
Male	8 (25.0)	24 (75.0)	
Female	15 (20.3)	59 (79.7)	
**Arterial hypertension**			0.500 ^ǂǂ^
No	19 (22.4)	66 (77.6)	
Yes	4 (19.1)	17 (80.9)	
**Diabetes Mellitus**			0.600 ^ǂǂ^
No	22(22.0)	78 (78.0)	
Yes	1 (16.7)	5 (83.3)	
**Insulin resistance**			0.500 ^ǂǂ^
No	4(22.2)	14 (77.8)	
Yes	19 (21.6)	69(79.4)	
***Helicobacter pylori***			0.600 ^ǂ^
No	16 (22.9)	54 (77.1)	
Yes	7 (19.4)	29 (80.6)	
**Anthropometric characteristics**			
BMI	36 [33-39]	38 [34-41]	0.260 ††
Abdominal circumference	107 [95-116]	113 [100-123]	0.120 ††
Body fat percentage	44 [35-48]	46 [37-55]	0.180 ††
**Clinical and laboratorial characteristics**			
SBP	124.82 ± 15.85	120.2 ± 14.3	0.190 †
DBP	79.82 ± 8.9	77.54 ± 10.02	0.330 †
Glucose	85 [81-92]	89 [81-95]	0.380 ††
Cholesterol	202 [172-228]	189 [164-227]	0.310 ††
HDL	45 [36-54]	46 [37-55]	0.820 ††
LDL	117 [109-132]	112 [91-136]	0.330 ††
VLDL	26 [19-37]	29 [21-37]	0.960 ††
Triglycerides	129 [96-185]	146 [104-185]	0.890 ††
Insulin	18 [14-28]	19 [14-39]	0.460 ††
HOMA-IR	4 [3-6]	4 [3-8]	0.450 ††

BMI body mass index, SBP systolic blood pressure, DBP diastolic blood pressure HDL high-density lipoprotein, LDL low-density lipoprotein, VLDL, very low-density lipoprotein, HOMA-IR homeostatic model assessment- insulin resistance

^†^ Student's t-test

^††^ Mann Whitney U

^ǂ^ Chí ^2^ test

^ǂǂ^ Fisher's exact test

On the multivariable analyses, after adjusting for age and sex, we found that a BMI ≥ 40 kg/m ^2^ (aRR = 0.59, 95% CI = 0.18-1.40), an elevated BFP (aRR = 0.41, 95% CI = 0.15-1.19) and the WC (aRR = 0.91, 95% CI = 0.69-1.16) were not associated with a higher risk of developing EE at three months after sleeve gastrectomy. Conversely, these high anthropometric indexes seemed to reduce the risk of *de novo* EE, but these results were not significant ( Table 4).

**Table 4.** Association between the body mass index, waist circumference, body fat mass and the development of esophagitis.

		Crude model **		Adjusted model **	
Variables		cRR	95% CI	aRR †	95% CI
Body mass index					
	< 40 kg/m ^2^	Ref.		Ref.	
	≥ 40 kg/m ^2^	0.64	0.20-1.46	0.59	0.18-1.40
Body fat mass (%)					
	Normal	Ref.		Ref.	
	Elevated	0.50	0.23-1.27	0.41	0.15-1.19
Waist circumference (cm) *		0.94	0.75-1.33	0.91	0.69-1.16

RR: relative risk CI: 95% Confidence Interval

* It was modeled by adding a quadratic variable.

** Non-parametric bias-corrected and accelerated bootstrap confidence interval estimation with 1000 replications for generalized linear models with Poisson link-log family and robust standard errors.

^†^ Adjusted by age and sex

## Discussion

In this study, we assessed the association between BMI, WC, and BFP and the development of *de novo* EE in adults with obesity three months after undergoing sleeve gastrectomy. Although none of the variables showed significant differences according to the development of *de novo* EE, we found that the female patients more frequently developed EE and patients without EE had higher anthropometric indexes. We did not find any association in crude and adjusted models between BMI, WC and BFP with the development of *de novo* EE.

Our study showed that male patients presented a higher BMI, WC, and BFP compared to women, results that are similar to previous literature. ^24^^–^^26^ The BMI represents the overall body mass that includes visceral and subcutaneous fat, muscle, bone, and major organs, among others. Due to genetic, environmental, and behavioral factors men tend to have a greater fat amount and distribution. ^27^ In addition, the sexual hormonal responses lead to obesogenic changes ^28^ and sexual dimorphism with a high impact in the WC. ^27^^,^^29^ However, Lihua *Hu et al.* found that women present a greater proportion of visceral fat than men, being the only anthropometric measurement that prevails between the two sexes, and this tends to increase over time. ^30^ High adiposity values in the abdominal circumference stimulates the release of fatty acids, thereby increasing the availability of glucose and hyperinsulinism ^31^^,^^32^ and favoring the development of T2DM due to low sensitivity of the glucose transporter receptors of the organs to insulin, ^33^ which may explain and correlate with the results described in our study.

Currently, there are several parameters for measuring obesity, and among these, the measurement of visceral body fat percentage by computed tomography is considered one of the best predictors of GERD. ^34^ However, the BMI, WC and the percentage of BFP are more accessible and less costly parameters to obtain. Several studies reported that an elevation of these anthropometric indexes was associated with the development of EE in bariatric surgery candidates. ^7^^,^^9^^,^^35^^,^^36^ In fact, previous studies have shown that men with higher obesity values ^37^ and the presence of hiatal hernia ^38^ are more likely to develop EE. However, in our study, firstly we excluded the patients with preoperative hiatal hernia in order to avoid this as a confounder, and regarding the anthropometric indexes, we did not find a significant association with de novo esophagitis, which could be explained by the fact that the EE group was composed of a higher proportion of women with lower-than-expected anthropometric indexes.

There are different pathophysiological mechanisms to explain how obesity can cause GERD. Previous studies have indicated that obesity may cause EE by mechanical factors such as high intra-abdominal and intragastric pressure, ^39^ an increased LES relaxation, a high gastroesophageal pressure gradient ^40^; as well as physiological factors such as increased bile and pepsin composition of gastric contents ^41^ and high leptin levels. ^42^ Sleeve gastrectomy is currently leading up to 80% of weight loss in a long-term setting. ^43^ A recent study demonstrated that a substantial reduction in BMI is required to induce the resolution of esophagitis, especially in individuals with obesity. Moreover, a study reported that the resolution rate was twice as high in subjects who achieved a BMI reduction of more than 2 kg/m ^2.^ ^44^ Nevertheless, in our study, we found that higher anthropometric indexes were not associated with the development of EE after sleeve gastrectomy. This could be explained by the fact that patients with higher levels of obesity tend to have a greater and more rapid weight loss compared to those with a lower BMI who tend to achieve a more sustained weight loss. Indeed, this suggests that a controlled reduction of the BMI by sleeve gastrectomy may constitute an effective measure to prevent the development of esophagitis by the alleviation and control of the pathophysiologic factors involved in this outcome.

To our knowledge, this is the first study to show that having a higher obesity index is not a risk factor for the development of *de novo* EE at three months post sleeve gastrectomy. However, the present study has some limitations. First, external validity is limited because the results come from a single center and are limited to adult patients with obesity. Second, the patients were followed for only three months, and thus, long-term results were not available. Third we could not include the assessment of proton bump inhibitor (PPI) use within the period of evaluation, however, according to clinic protocol, lansoprazole 30 mg is indicated for the first postoperative month and omeprazole 20 mg for the following two months, and depending on the reflux symptoms after three months of treatment, the use of PPIs can be postponed. Finally, in spite of the usage of the bioelectrical impedance scale for the measurement of BFP, a computed tomography would have been better to measure the visceral BFP as it has been reported to be more accurate as a predictor of EE according to the literature.

## Conclusion

In conclusion, there were no significant differences between anthropometric indexes and the development of *de novo* EE esophagitis at three months post sleeve gastrectomy. Based on these results, we consider that morbid obesity should not be an excluding factor for undergoing sleeve gastrectomy as the surgery of choice for weight loss because of the risk of developing EE. Nonetheless, further studies are needed to evaluate this association in gastrectomized patients over a longer follow-up period.

## Data availability statement

### Undelying data

Harvard Database: Association between the body mass index, waist circumference, and body fat percentage with erosive esophagitis in adults with obesity after sleeve gastrectomy. https://doi.org/10.7910/DVN/ZBVTY4

This project contains the following files:

• Association between the body mass index, waist circumference, and body fat percentage with erosive esophagitis in adults with obesity after sleeve gastrectomy.tab (raw data file)
• README_Association between the body mass index, waist circumference, and body fat percentage with erosive esophagitis in adults with obesity after sleeve gastrectomy.txt (data key)

Data are available under the terms of the Creative Commons Zero "No rights reserved" data waiver (CC0 1.0 Public domain dedication).
