# Supplementary material for: Association between the body mass index, waist circumference, and body fat percentage with erosive esophagitis in adults with obesity after sleeve gastrectomy
Source: F1000Res. 2022 Jun 1;11:214. Originally published 2022 Feb 22. [Version 2] doi: 10.12688/f1000research.106723.2 (PMC9535328; doi:10.12688/f1000research.106723.2)
Supplement: Peer review history

# Peer review: Reviewer response for version 2

**DOI:** 10.5256/f1000research.134543.r150289

**Published:** 2022-10-05

**Authors:** Moisés Apolaya-Segura

**Affiliations:** 1 Facultad de Medicina, Universidad de San Martín de Porres, Chiclayo, Peru

The research allows evaluating the main biophysical variables and their association with the incidence of erosive esophagitis in an obese population after sleeve gastrectomy; it uses adequate selection criteria, being the preoperative evaluation a strength. However, it is necessary to add in the discussion aspects related to the short follow-up time carried out in the present study, with respect to other studies that have a longer follow-up time, and to discuss the postoperative treatment received, such as proton pump inhibitors, because perhaps these drugs can explain the results found (non-association), since in the limitations it is understood that the patients received as protocol the use of proton pump inhibitors is used for 2 to 3 months after surgery. Therefore, the conclusion should be conservative, until studies are developed with a longer follow-up time and consider the postoperative treatment received.

Is the work clearly and accurately presented and does it cite the current literature?

Yes

If applicable, is the statistical analysis and its interpretation appropriate?

Yes

Are all the source data underlying the results available to ensure full reproducibility?

Partly

Is the study design appropriate and is the work technically sound?

Yes

Are the conclusions drawn adequately supported by the results?

Yes

Are sufficient details of methods and analysis provided to allow replication by others?

Partly

Reviewer Expertise:

epidemiology and public health physician

I confirm that I have read this submission and believe that I have an appropriate level of expertise to confirm that it is of an acceptable scientific standard, however I have significant reservations, as outlined above.

# Peer review: Reviewer response for version 1

**DOI:** 10.5256/f1000research.117659.r135643

**Published:** 2022-04-25

**Authors:** Kei Nakajima

**Affiliations:** 1 School of Nutrition and Dietetics, Faculty of Health and Social Services, Kanagawa University of Human Services, Yokosuka, Japan

This study aims to assess the association between the body mass index (BMI), waist circumference (WC), and body fat percentage (BFP) with the development of EE in adults with obesity three months after SG in a retrospective cohort of 106 obese patients. In conclusion, the authors found no association between preoperative anthropometric indexes and the development of de novo EE. This article is well written and of clinical interest. However, I have some concerns.

**Major comments:**

1. It is unclear why the authors selected the time point of three months after the surgery. I wonder if gastric function does not reach full recovery at the time point.
2. The primary purpose of sleeve gastrectomy is for weight loss. However, such data is not provided in this study. It may be interesting if weight loss is associated (inversely?) with the incidence of erosive esophagitis.
3. I suggest that the reference of BMI should be less than 30 or 35 in the regression model. In addition, I would like to know whether a dose effect association exists after classification into three or four groups, for instance, <30, 30-35, 35-40, > 40 BMI.
4. It may be better to include helicobacter pylori infection as a confounding factor in the logistic regression model.

Is the work clearly and accurately presented and does it cite the current literature?

No

If applicable, is the statistical analysis and its interpretation appropriate?

Partly

Are all the source data underlying the results available to ensure full reproducibility?

No

Is the study design appropriate and is the work technically sound?

Yes

Are the conclusions drawn adequately supported by the results?

Partly

Are sufficient details of methods and analysis provided to allow replication by others?

No

Reviewer Expertise:

Obesity, diabetes, nutrition, cardiovascular risk factors

I confirm that I have read this submission and believe that I have an appropriate level of expertise to confirm that it is of an acceptable scientific standard, however I have significant reservations, as outlined above.

# Response

**Published:** 2022-05-27

**Authors:** Alba Zevallos

**Affiliations:** Universidad Cientifica del Sur, Peru

Thank you for the review of our manuscript. We would like to express our gratitude for your time invested in reading the paper. We are also thankful for the comments. Please, find our point-by-point response to the comments provided below. All changes made are included in the new version of the manuscript.

**R2C1: **It is unclear why the authors selected the time point of three months after the surgery. I wonder if gastric function does not reach full recovery at the time point.

**AR1: **Thank you so much for the comment. We would have liked to evaluate a longer follow-up; however, we have limited data to perform this analysis. Furthermore, we considered evaluating erosive esophagitis at three months cause the study by Jan.S Burgerhart showed an increase in acid exposure time at 3 months post sleeve gastrectomy measured by manometry, which is why we consider that there could be endoscopic signs of erosive esophagitis as early as 3 months post sleeve gastrectomy. We have added this information in the introduction: *“* *Jan S. Burgerhart showed that* *esophageal acid exposure increased significantly when comparing 24-h pH measurements before and 3 months after sleeve gastrectomy.* *[15]* *”*

**R2C2: **The primary purpose of sleeve gastrectomy is for weight loss. However, such data is not provided in this study. It may be interesting if weight loss is associated (inversely?) with the incidence of erosive esophagitis.

**AR2: **Thank you very much for the comment. We have added information about the weight loss in the results section: “ *A higher mean of weight loss at 3 months postoperatively was observed in morbidly obese patients (26.78 kg vs. 19.50 kg).”* We agree that it would be interesting to evaluate whether weight loss is associated with the development of erosive esophagitis, however, that is beyond the scope of our study.

**R2C3: **I suggest that the reference of BMI should be less than 30 or 35 in the regression model. In addition, I would like to know whether a dose effect association exists after classification into three or four groups, for instance, <30, 30-35, 35-40, > 40 BMI.

**AR3: **Thank you very much for your suggestion, we will consider the new cut-off point for BMI for future studies. On the other hand, we believe that polytomizing the BMI variable would result in a loss of statistical power since the number of patients enrolled is not very large.

**R2C4: **It may be better to include helicobacter pylori infection as a confounding factor in the logistic regression model.

**AR4: **Thank you for the suggestion. According to our DAG (directed acyclic graph), the H. pylori infection was not considered as a confounder. We do believe that H. pylori is associated with obesity; however, it is important to mention that all our study participants had obesity. In addition, we had to mention that, since we only had 23 events, adding one more variable to our regression model could have generated overfitting.

# Peer review: Reviewer response for version 1

**DOI:** 10.5256/f1000research.117659.r124758

**Published:** 2022-03-11

**Authors:** Sergio Goicochea-Lugo

**Affiliations:** 1 EsSalud, Instituto de Evaluación de Tecnologías en Salud e Investigación, Lima, Peru

Congratulations for the article, it is important to be able to identify potential risk factors associated with erosive esophagitis, even more so, using a design that can estimate relative risks.

 I would also like to make the following comments that I hope will be useful.

**Regarding the methods section:**

 I would recommend specifying the moment in which the data of the body mass index, waist circumference, and body fat percentage were obtained since it was not clear to me if these values ​​were obtained before performing the gastrectomy (and at what time) or were obtained after performing the gastrectomy (and at what time).

 This seemed important to clarify the discussion since you mention that not finding statistical differences could be due to the fact that the patients have a rapid and progressive weight loss. If it is possible to show the data of these variables three months after surgery in a table or as a narrative part, it would support this hypothesis.

**Regarding the statistical analyses:**

 In the bivariate analyses, you found statistical differences between men and women. Have you raised the possibility that sex is an effect modifier rather than a confounder? It could be important to carry out an analysis with the regression model for men and women separately, taking into account the statistical and biological basis that you comment on in the discussion.

 Other than that, I found the limitations mentioned and how they were dealt with to be correct.

Is the work clearly and accurately presented and does it cite the current literature?

Yes

If applicable, is the statistical analysis and its interpretation appropriate?

Partly

Are all the source data underlying the results available to ensure full reproducibility?

Yes

Is the study design appropriate and is the work technically sound?

Yes

Are the conclusions drawn adequately supported by the results?

Yes

Are sufficient details of methods and analysis provided to allow replication by others?

Yes

Reviewer Expertise:

evidence based medicine

I confirm that I have read this submission and believe that I have an appropriate level of expertise to confirm that it is of an acceptable scientific standard.

# Response

**Published:** 2022-05-27

**Authors:** Alba Zevallos

**Affiliations:** Universidad Cientifica del Sur, Peru

Thank you for the review of our manuscript. We would like to express our gratitude for your time invested in reading the paper. We are also thankful for the comments. Please, find our point-by-point response to the comments provided below. All changes made are included in the new version of the manuscript.

**R1C1:** I would recommend specifying the moment in which the data of the body mass index, waist circumference, and body fat percentage were obtained since it was not clear to me if these values ​​were obtained before performing the gastrectomy (and at what time) or were obtained after performing the gastrectomy (and at what time). This seemed important to clarify the discussion since you mention that not finding statistical differences could be due to the fact that the patients have a rapid and progressive weight loss. If it is possible to show the data of these variables three months after surgery in a table or as a narrative part, it would support this hypothesis.

**AR1: **Thank you very much for the comment. We have added the time the anthropometric indexes were measured in the methods section: “ *All three anthropometric indexes were measured on the day of surgery.*” Moreover, added information about the weight loss in the results section: “A higher mean of weight loss at 3 months postoperatively was observed in patients with morbid obesity (26.78 kg vs 19.50 kg).”

**R1C2:** In the bivariate analyses, you found statistical differences between men and women. Have you raised the possibility that sex is an effect modifier rather than a confounder? It could be important to carry out an analysis with the regression model for men and women separately, taking into account the statistical and biological basis that you comment on in the discussion.

**AR2: **Thank you for your comment. We agree that a sex-stratified analysis would have been interesting. Unfortunately, due to our limited sample size, we consider that this would have affected our estimates (and our confidence intervals). However, we hope to consider these analyses in further studies.
